# Root extract of water dropwort, *Oenanthe javanica* (Blume) DC, induces protein and gene expression of phase I carcinogen-metabolizing enzymes in HepG2 cells

**DOI:** 10.1186/s40064-016-2078-8

**Published:** 2016-04-06

**Authors:** Jae Kyeom Kim, Eui-Cheol Shin, Gwi Gun Park, Youn-Jung Kim, Dong-Hoon Shin

**Affiliations:** School of Human Environmental Sciences, University of Arkansas, 118 HOEC, Fayetteville, AR 72701 USA; Department of Food Science, Gyeongnam National University of Science and Technology, Chilam-dong, Jinju, 660-758 Republic of Korea; Department of Food Science and Biotechnology, Gachon University, Seongnam, 461-701 Republic of Korea; College of Nursing Science, Kyunghee University, Seoul, 136-701 Republic of Korea; Department of Food and Biotechnology, Korea University, Anam-dong, Seongbuk-gu, Seoul, 136-701 Republic of Korea

**Keywords:** Phase I enzymes, Cytochrome P450, Xanthotoxin, Bergapten, Isopimpinellin

## Abstract

**Background:**

Cytochrome P450 (CYP) isoenzymes are an important phase I enzyme system. In the present study, we investigated the effects of *Oenanthe javanica* (Blume) DC on CYP1A1 and CYP1A2.

**Findings:**

Whole plants were completely dried and then divided into leaves, stems, and roots for extraction. The human liver hepatocellular carcinoma cell line HepG2 was treated with ethanol extracts of these organs for 72 h and mRNA and protein expression levels were assessed. The root extract of *O. javanica* significantly elevated the expression of both CYP1A1 and CYP1A2 mRNAs (by 68 and 102 %, respectively). Similarly, the CYP1A1 and CYP1A2 protein levels were increased by the root extract (by 112 and 157 %, respectively). The effects of the root extract were much more pronounced than those of leaf and stem extracts. Subsequent GC–MS analysis revealed that the levels of major coumarin derivatives, xanthotoxin, bergapten, and isopimpinellin, were significantly higher in *O. javanica* root extracts than in leaf or stem extracts. Of note, 5 μM xanthotoxin (the most abundant furanocoumarin in *O. javanica*) induced the expression of CYP1A1 mRNA as well as CYP1A2 mRNA and protein, albeit the CYP1A1 protein level was elevated only at 10 μM xanthotoxin.

**Conclusions:**

Although it is difficult to extrapolate such effects to metabolic outcomes because of the inherent limitations of in vitro experiments, it is important to note that dietary exposure to *O. javanica* may modulate phase I enzymes and thereby affect various xenobiotic metabolism.

**Electronic supplementary material:**

The online version of this article (doi:10.1186/s40064-016-2078-8) contains supplementary material, which is available to authorized users.

## Background

Humans are constantly exposed to a variety of foreign chemicals, often called xenobiotics, and thus have a few defense systems against these compounds. Biotransformation enzymes are part of these defense systems; these enzymes play a critical role in metabolism, elimination, and detoxification of xenobiotics and endogenous compounds (Xu et al. 2005). Among the biotransformation enzymes, cytochrome P450 (CYP) isoenzymes are an important phase I enzyme system as they add functional groups to parent compounds to produce more polar metabolites (Liska 1998). CYPs are found in nearly all organisms from bacteria to humans and are expressed in various tissues, mainly in the liver, small intestine, kidney, and lungs (Myasoedova 2008; Patterson and Murray 2002). Even though most CYP-mediated oxidation reactions are considered as detoxification, a number of pro-carcinogens can be metabolically activated by these enzymes, which elicit their mutagenic and carcinogenic potentials. Specifically, CYP1As are able to activate dietary pro-carcinogens (Eaton et al. 1995), emphasizing the significance of a interaction between dietary chemicals and the function of phase I enzymes.

*Oenanthe javanica* (Blume) DC (also known as water dropwort) is an aquatic perennial plant of the *Apiaceae* family and widely consumed as a spicy vegetable in East Asian countries. Multiple studies have reported health-promoting effects of *O. javanica* in different experimental models, including anti-oxidative and anti-mutagenic activity in colon cancer cells (Kwon et al. 2006), an antiviral effect (Wang et al. 2005), and hepato-protective activity in vitro and in vivo (Yang et al. 2014). Of note, Kim et al. (2009) have demonstrated that the butanol fraction of *O. javanica* accelerates ethanol metabolism in vivo, indicating that *O. javanica* extracts may modulate biotransformation enzymes responsible for xenobiotics metabolism (e.g., CYP2E1). However, to the best of our knowledge, no studies have compared the potential of different parts of *O. javanica* to stimulate the activity of phase I biotransformation enzymes or identified the active constituents. Therefore, in the present study, we compared the effects of leaf, stem, and root extracts of *O. javanica* on mRNA and protein expression of CYP1A1 and CYP1A2, and demonstrated that a major coumarin derivative might be responsible, at least in part, for these effects.

## Methods

### Materials

All chemicals were obtained from Sigma-Aldrich Co. (St. Louis, MO, USA), unless otherwise specified. The HepG2 cell line was purchased from the American Type Culture Collection (HP 8065; Manassas, VA, USA). *O. javanica* was obtained from a local market (Jinju, Republic of Korea). Upon purchase, the identity of *O. javanica* was confirmed and a specimen voucher was issued by the Department of Agriculture and Herbal Resources of Gyeongnam National University of Science and Technology, South Korea (voucher number: GFA-088).

### Sample preparation and extraction

Whole plants were completely dried at room temperature and divided into leaves, stems, and roots. Detailed procedures are described in the Additional file 1: Supplemental Methods.

### Cell culture and sample treatments

HepG2 cells were cultured in Dulbecco’s Modified Eagle Media. Detailed experimental conditions for cell culture and sample treatments are provided in the Additional file 1: Supplemental Methods.

### Measurement of cell viability

A conventional MTT assay was performed to evaluate cytotoxicity of *O. javanica* extracts as we described (Kim et al. 2013). Extract concentrations ranged from 100 to 1600 μg/mL and pretreated to the cells for 48 h.

### Western blot analysis

CYP1A1 and CYP1A2 protein expression was measured using Western blot analysis. Please refer to the Additional file 1: Supplemental Methods for further details.

### Real-time RT-PCR analysis

Total RNA was extracted from HepG2 cells using the RNeasy Mini kit according to the manufacturer’s instructions (Qiagen, Hilden, Germany). The A260/A280 ratios of all RNA samples were in the range of 1.8–2.0. Detailed experimental conditions for reverse transcriptase reactions and quantitative real-time RT-PCR analysis are provided in the Additional file 1: Supplemental Methods.

### GC–MS analysis

To quantify candidate active constituents present in *O. javanica*, leaf, stem, and root extracts were derivatized and then subjected to GC–MS analysis as described elsewhere (Kim et al. 2014).

### Statistical analysis

All data were expressed as mean ± SEM. The statistical significance of the differences between the groups was calculated by one-way analysis of variance, followed by the Duncan’s multiple-range test (SAS ver. 9.0; SAS Institute, Cary, NC, USA). *P* values less than 0.05 were considered to be statistically significant.

## Results and discussion

**Fig. 1:**
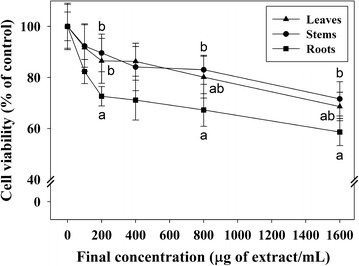
The effects of *O. javanica* extracts on HepG2 cell viability. Cell viability was assessed by the conventional MTT method and expressed as a percentage of that of DMSO-treated control cells. Extract concentrations ranged from 100 to 1600 μg/mL. Data were expressed as mean ± SEM (*n* = 3). The statistical significance of the differences between the samples (i.e., leave stem and root extracts) was calculated by one-way analysis of variance, followed by the Duncan’s multiple-range test. Values labeled with the *same superscript letters* are not significantly different

Overall, HepG2 cell viability gradually decreased with increasing concentrations of *O. javanica* extracts (Fig. 1). The effect of the root extract on cell viability was more pronounced than those of the other two samples. In particular, compared to stem extract treatment, the viability of HepG2 cells was significantly lower after treatment with 200, 800, and 1600 μg of root extracts/mL (89.6 ± 7.4 vs. 72.6 ± 3.8, 83.0 ± 5.7 vs. 67.2 ± 6.4, and 71.6 ± 6.7 vs. 58.6 ± 5.3, respectively). No difference in cell viability was noted between stem and leaf extract treatments within the range of concentrations tested (Fig. 1).

The levels of CYP1A1 and CYP1A2 transcripts were significantly elevated in response to treatments with root extract (100 µg of extract/mL concentration) of *O. javanica* (68 and 102 % increase compared to the DMSO treated group, respectively; Table 1). In a similar manner, Western blot analysis demonstrated that the levels of the CYP1A1 and CYP1A2 proteins were significantly increased (2.1- and 2.6-fold, respectively) after treatment with root extracts in comparison with control treatment. Treatments with leaf and stem extracts did not show similar potency in increasing the transcript and protein levels of these enzymes (Table 1).

**Table 1:** Changes in gene and protein expression of CYP1A1 and CYP1A2 induced by water dropwort extracts

	CYP1A1		CYP1A2	
	Gene expression (fold change)	Protein expression (fold change)	Gene expression (fold change)	Protein expression (fold change)
Control	1.00 ± 0.14^a^	1.00 ± 0.17^a^	1.00 ± 0.09^a^	1.02 ± 0.19^a^
Leaf extract	1.02 ± 0.15^a^	1.31 ± 0.12^a^	1.14 ± 0.20^a^	1.27 ± 0.12^a^
Stem extract	1.22 ± 0.16^ab^	1.25 ± 0.17^a^	1.38 ± 0.21^ab^	1.42 ± 0.15^a^
Root extract	1.68 ± 0.18^b^	2.12 ± 0.16^b^	2.02 ± 0.13^b^	2.57 ± 0.14^b^

HepG2 cells were treated with either *O. javanica* extracts (100 µg of extract/mL final concentration) or vehicle control (DMSO, 0.5 % final concentration, v/v) for 72 h and harvested for analyses of mRNA and protein expression. Data were expressed as mean ± SEM (*n* = 3). The statistical significance of the differences between the samples was calculated by one-way analysis of variance, followed by the Duncan’s multiple-range test. Changes are indicated relative to DMSO treated controls. Values labeled with the same superscript letters within a column are not significantly different

**Fig. 2:**
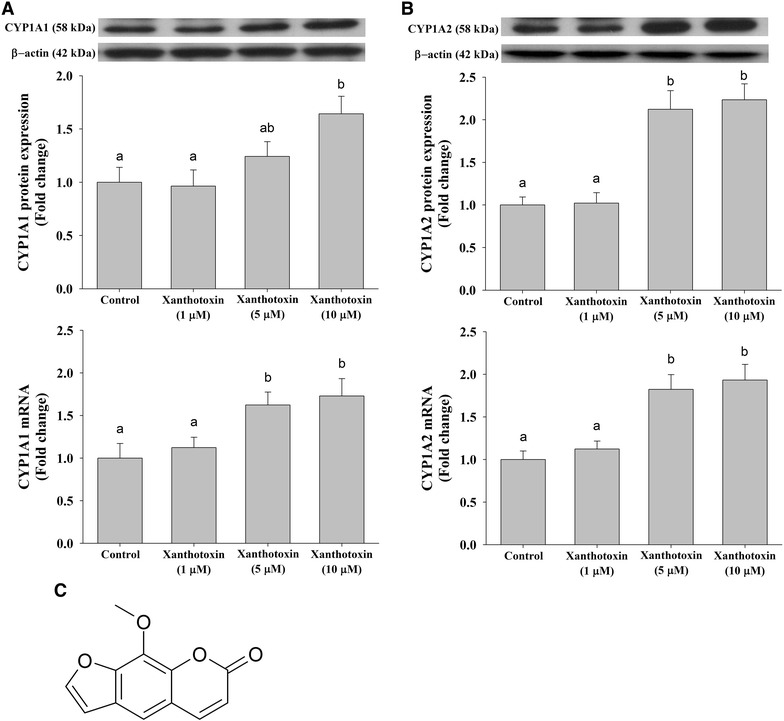
The effects of xanthotoxin from *O. javanica* extracts on CYP1A1 and CYP1A2 mRNA and protein expression in HepG2 cell viability. Effects on treatments of xanthotoxin on protein and mRNA expression of CYP1A1 (**A**) and CYP1A2 (**B**) were assessed. The chemical structure of xanthotoxin is provided (**C**). Data were presented as fold changes relative to the control group (i.e., DMSO treated control cells) and expressed as mean ± SEM (*n* = 3). The statistical significance of the differences was calculated by one-way analysis of variance, followed by the Duncan’s multiple-range test. Values labeled with the *same superscript letters* are not significantly different

To elucidate the candidate compounds responsible for such modulation in vitro, different parts of *O. javanica* were subjected to GC–MS analysis. Given that the *Apiaceae* are a rich source of furanocoumarins [e.g., up to 77 % of phytochemical contents (Autore et al. 2015)], we specifically targeted to measure three major furanocoumarins (xanthotoxin, bergapten, and isopimpinellin). Interestingly, we found that the contents of these three compounds were noticeably higher in the root extract of *O. javanica* than in the leaf and stem extracts. The levels of xanthotoxin in leaf, root and stem extracts were 70.9 ± 6.4, 164.6 ± 12.1, and 71.3 ± 8.1 μg/g dry weight, respectively. Similarly, the level of isopimpinellin was highest in the root extract (17.6 ± 1.3 μg/g dry weight), followed by the stem (12.7 ± 2.0 μg/g dry weight) and leaf extracts (5.8 ± 0.8 μg/g dry weight). Lastly, the content of bergapten in the root extract was 61.1 ± 3.7 μg/g dry weight, yet bergapten was not detected in either leaves or stems of *O. javanica*. On the basis of these stark differences in furanocoumarin contents, we hypothesized that one of these compounds is responsible for the induction of CYP1A1 and CYP1A2 mRNA and protein expression. Thus, the most abundant furanocoumarin, xanthotoxin, was further tested with regards to its effects on CYP1A1 and CYP1A2. Interestingly, we found a dose-dependent induction of both CYP1A1 (Fig. 2A) and CYP1A2 (Fig. 2B) mRNA and protein expression.

Individual furanocoumarins have received great attention due to their wide range of biological impacts including beneficial effects in psoriasis and vitiligo treatments (Linden and Weinstein 1999; Matin 2011), inhibition of cholinesterase activity in mice brain (Kim et al. 2011), and anti-bacterial and anti-malarial activity (Wangchuk et al. 2014). However, only a few studies have investigated their effects on biotransformation enzymes and such data are somewhat limited and inconsistent. For instance, it was previously reported that CYP1A2 activity might be inhibited by bergapten (Bendriss et al. 1996) and xanthotoxin (Mays et al. 1987). In another study, 1 nM to 1 mM xanthotoxin and isopimpinellin decreased CYP1A1 activity in rat hepatocytes and isolated microsomes, ultimately resulting in complete inhibition (Baumgart et al. 2005). However, xanthotoxin and angelicin increased the CYP1A1 mRNA levels at high concentrations (i.e., >100 μM). Similarly, Gwang demonstrated a biphasic effect of xanthotoxin on CYP1A1 and CYP1A2 activities, which were repressed by 18 and 17 % after 2 h of xanthotoxin treatment and remarkably elevated by 727 and 932 % after 24 h, respectively (Gwang 1996). Such biphasic effects indicate that furanocoumarins may act as both inducers and inhibitors of these enzymes. Under our experimental conditions, however, the expression of CYP1A1 and CYP1A2 mRNAs and CYP1A2 protein was significantly increased at 5 μM xanthotoxin (Fig. 2), albeit CYP1A1 protein expression was elevated only at 10 μM of xanthotoxin after 72 h treatment. On the other hand, when this compound was orally administered in human (1 mg/kg body weight), the plasma concentration of xanthotoxin was shown to be the highest after 2 h while the concentration was approximately 1.6 ng/mL (Brautigam et al. 2003). Although it is difficult to extrapolate such effects to metabolic outcomes because of the inherent limitations of in vitro experiments, it is important to note that dietary exposure to *O. javanica* may modulate phase I enzymes and thereby affect various xenobiotic metabolism. Another potential limitation is that increase in gene and protein expressions may always correlate with enzymatic activities. This is because even though, as we demonstrated herein, bio-actives (e.g., xanthotoxin) in vegetables act as an inducer of certain genes, some constituents may either inhibit or activate these enzymes through regulatory mechanisms other than transcriptional regulation [e.g., allosteric activation; (Ludwig et al. 1999)]. Last, we only assessed the effects of xanthotoxin in the study despite that bergapten and isopimpinellin may elicit similar biological effects, at least in some extent. However, xanthotoxin was chosen for further analyses because (1) it was the most mass abundant compound, by far, compared to the other two derivatives, (2) bergapten was not even detected in leaves and stems of *O. javanica*, and (3) our main objective was to pinpoint the compound, responsible for impacts of water dropwort. In addition, due to the structural similarities in these derivatives, it is difficult to believe that effects of the other two furanocoumarins would exceed the effects of xanthotoxin given approximately tenfold difference in weight between compounds (e.g., xanthotoxin vs. isopimpinellin; 164.6 ± 12.1 vs. 17.6 ± 1.3 μg/g dry weight of roots).

Collectively, to the best of our knowledge, this is the first study that (1) compared the effects of leaf, stem, and root extracts of *O. javanica* on mRNA and protein expression of CYP1A1 and CYP1A2, and (2) demonstrated that xanthotoxin, the major coumarin derivative present in the root extract of *O. javanica*, might be responsible, at least in part, for these effects. Therefore, this study provides interesting preliminary data for future in vivo and human studies aimed at elucidation of the metabolic impact of chronic or short-term dietary consumption of *O. javanica*. In addition, given the potential roles of these CYP1As in carcinogen metabolism (Eaton et al. 1995), further investigations of the cancer-preventive potential of *O. javanica* might also be warranted.

## Additional file

10.1186/s40064-016-2078-8 Detailed experimental procedures and conditions are described in the file for readership.

## Abbreviation

CYP: cytochrome P450
