# Supplementary material for: Root extract of water dropwort, Oenanthe javanica (Blume) DC, induces protein and gene expression of phase I carcinogen-metabolizing enzymes in HepG2 cells
Source: Springerplus. 2016 Apr 6;5:413. doi: 10.1186/s40064-016-2078-8 (PMC4821844; doi:10.1186/s40064-016-2078-8)
Supplement: Supplementary file 1 — 10.1186/s40064-016-2078-8 Detailed experimental procedures and conditions are described in the file for readership. [file 40064_2016_2078_MOESM1_ESM.docx]

**Supplemental Methods**

**Sample Preparation and Extraction**

Whole plants were completely dried at room temperature and divided into leaves, stems, and roots. Dried materials were ground and extracted with 10 volumes of ethanol in 125 mL flasks. Samples were stirred at 150 rpm (C-Ski-II; YS-Tech Co., Seoul, South Korea) overnight and then filtered through a Whatman no. 42 ashless filter (Whatman International Co., Kent, UK). The residue was extracted with the same volume of fresh ethanol once more. The supernatants were combined and evaporated under reduced pressure at 40°C. Completely dried extracts were weighed and reconstituted using 5% DMSO in water (v/v) and stored at −80°C until analysis.

**Cell Culture and Sample Treatments**

HepG2 cells were maintained in Dulbecco’s Modified Eagle Media (Cat #: **D5546**; Sigma-Aldrich; St. Louis, MO, USA) containing 10% heat-inactivated fetal bovine serum (Cat #: **F6178**; Sigma), 20 μM L-glutamine (Cat #: **G7513**, Sigma) and 10 IU/mL penicillin, 10 μg/mL streptomycin, and 25 ng/mL amphotericin B (Cat #: 15-240-112, Gibco^TM^; Thermo Fisher Scientific, Waltham, MA, USA) in an incubator at 37°C with water saturation at 5% CO_2_. The cells were passaged when 80–90% confluent. The medium was changed at least three times a week. To investigate the effects of *O. javanica* extracts and their active constituents, 5 × 10^5^ cells were seeded per culture dish and grown as above for 24 h. Cells were then treated with either samples or vehicle control (DMSO, 0.5% final concentration, v/v) for 72 h and harvested for analyses of mRNA and protein expression.

**Western blot analysis**

CYP1A1 and CYP1A2 protein expression was measured using Western blot analysis. After treatments, harvested cells were homogenized in ice-cold lysis buffer (50 mM Tris-HCl containing 150 mM NaCl, 1% Nonidet P-40, 0.1% SDS, 1 mM EDTA, 1 mM phenylmethanesulfonyl fluoride, 1× Roche Complete Mini Protease Inhibitor Cocktail, and Roche PhosSTOP Phosphatase Inhibitor Cocktail). Homogenates were centrifuged at 10,000 × *g* for 30 min at 4°C and the supernatants were used for the analysis. Protein concentration was determined by the Bradford method (Bio-Rad Laboratories, Hercules, CA, USA). Equal amounts of protein were dissolved in 1× sample buffer containing 1% SDS (final concentration, v/v; Bio-Rad Laboratories), boiled for 10 min, and separated by 15% SDS-gel electrophoresis. Protein bands were transferred to polyvinylidene fluoride membranes. The membranes were incubated with blocking buffer [5% bovine serum albumin in Tris-buffered saline solution (0.5 M Tris base, 9% NaCl, and 1% Tween 20; pH 8.4)] for 2 h. Subsequently, membranes were incubated with primary antibodies against CYP1A1 (rabbit polyclonal IgG, Cat #: SC-20772), CYP1A2 (rabbit polyclonal IgG, Cat #: SC-30085), or β-actin (rabbit polyclonal IgG, Cat #: SC-130657, Santa Cruz Biotechnology, Dallas, TX, USA; 1:2000 dilution in the blocking buffer for all antibodies) overnight at room temperature and then with horseradish peroxidase–conjugated secondary antibody (goat anti-rabbit IgG-HRP, Cat #: SC-2004, Santa Cruz Biotechnology; 1:4000 dilution) for 2 h at room temperature. The blots were visualized with enhanced chemiluminescence solution (ImmunoCruz; Santa Cruz Biotechnology) and developed films were analyzed using the ImageJ software (National Institutes of Health, Bethesda, MD, USA).

**Real-time RT-PCR analysis**

Total RNA was extracted from HepG2 cells using the RNeasy Mini kit according to the manufacturer’s instructions (Qiagen, Hilden, Germany). The A260/A280 ratios of all RNA samples were in the range of 1.8 to 2.0. Reverse transcriptase reactions were done with SuperScript III First-strand Synthesis SuperMix (Invitrogen, Carlsbad, CA, USA) according to the manufacturer’s instructions. Quantitative real-time RT-PCR analysis was conducted using the ABI7500 real-time PCR system with SYBR green PCR Master Mix (Applied Biosystems, Foster City, CA, USA). Sequences of primers used for mRNA quantification were as follows: β-actin, 5′-ATTGCCGACAGGATGCAGA-3′ and 5′-GCTCAGGAGGAGCAATGATCTT-3′; CYP1A1, 5′-GTCATCTGTGCCATTTGCTTTG-3′ and 5′-CAACCACCTCCCCGAAATTATT-3′, and CYP1A2, 5′-TCCCCTCACACTTGTGTTCTC-3′ and 5′-AGGACTTCCCCGATACACCG-3′. Specific amplification of target sequences was verified by melt-curve analysis and the levels of CYP1A1 and CYP1A2 mRNAs were normalized to that of β-actin. Data were analyzed using the ΔΔC_T_ method and presented as fold changes relative to the control group (i.e., DMSO treated group). All analyses were run in triplicate.
